# Neurobiology of Major Depressive Disorder

**DOI:** 10.1155/2013/873278

**Published:** 2013-10-09

**Authors:** Rosa Villanueva

**Affiliations:** Servicio de Psiquiatría, Hospital Universitario La Paz, 28046 Madrid, Spain

## Abstract

We survey studies which relate abnormal neurogenesis to major depressive disorder. Clinically, descriptive gene and protein expression analysis and genetic and functional studies revised here show that individual alterations of a complex signaling network, which includes the hypothalamic-pituitary-adrenal axis; the production of neurotrophins and growth factors; the expression of miRNAs; the production of proinflammatory cytokines; and, even, the abnormal delivery of gastrointestinal signaling peptides, are able to induce major mood alterations. Furthermore, all of these factors modulate neurogenesis in brain regions involved in MDD, and are functionally interconnected in such a fashion that initial alteration in one of them results in abnormalities in the others. We highlight data of potential diagnostic significance and the relevance of this information to develop new therapeutic approaches. Controversial issues, such as whether neurogenesis is the basis of the disease or whether it is a response induced by antidepressant treatments, are also discussed.

## 1. Introduction

Major depressive disorder (MDD) is one of the most common psychiatric diseases. MDD is not only characterized by profound dysregulation of affect and mood but is also associated with other abnormalities including cognitive dysfunction, sleep and appetite disturbance, fatigue, and many other metabolic, endocrine, or inflammatory alterations (see [1, 2]). The existence of MDD as a medical condition has been recognized with the term *melancholia* in texts dating up to the ancient Greece, but the current diagnostic criteria remain to some degree arbitrary. In addition, account must be taken of the fact that almost all individuals have experienced a transient depressed mood state at some time in their life. In fact, there is controversy in whether MDD is best conceptualized as a disease or as the extreme of a continuum of increasingly disturbed affective regulation. MDD is often termed unipolar depressive disorder to be distinguished from depression which alternates with episodes of mania which is termed bipolar depression. The latter is potentially distinguishable by functional neuroimaging approaches [3].

The purpose of this review is to summarize information accumulated in the last two decades concerning gene and protein expression changes in MDD [4]. These data suggest that the pathophysiology of this disease is related to disturbed adult neurogenesis [5, 6] and, without doubt, will help develop new therapeutic and diagnostic tools in the near future [2, 7]. Due to the complexity of the subject, we will exclude from this review well-established monoamine neurochemical alterations in MDD which are the basis for most current treatments [8]. Anatomical identification of the brain regions altered in MDD has also advanced in the last decade with the employment of modern functional neuroimaging techniques (see [9]), but a detailed analysis of the anatomy and histopathology of the disease is also out of the scope of the present review.

Adult neurogenesis is a topic of increasing interest in neuroscience. In the last decadeit has been shown that, rather than being architecturally stable, the mammalian central nervous system retains potential to remove neurons and glia, and to establish new neural circuits. Two major cerebral zones, the subventricular region of the lateral ventricles, and the subgranular region of the gyrus dentatus, contain proliferating neural precursors able to provide neurons to be functionally integrated into neuronal networks. Neurogenesis in the sub-ventricular region provides neurons to the olfactory bulbus and is functionally implicated in olfaction. Adult born neurons produced in the gyrus dentatus are involved in major hippocampal functions and appear to be the target of diseases which impair memory and learning [10].

The etiology of depression is unknown (see review by [11]). MDD can be spontaneous but often follows a traumatic emotional experience or can be a symptom of other diseases, most often neurological (i.e., stroke, multiple sclerosis, or Parkinson disease) or endocrine (Cushing's disease, hypothyroidism). MDD can also be triggered or precipitated by pharmacological agents or drug abuse [12]. The prevalence is higher in woman (in the range of 1.5 to 2.5) and nearly 50% of the risk for depression is due to genetic factors [13]. These factors may influence both overall risk of illness and the sensitivity of individuals to the environmental adversities.

## 2. Neurotrophins and Depression

Histological and functional neuroimaging studies revealed synaptic and structural plasticity alterations in different regions of the brain, including the frontal cortex and hippocampus in MDD patients [14–17]. In pathophysiological terms, it was proposed that these alterations could prevent the brain from making appropriate adaptive responses to environmental stimuli [18]. These facts have directed attention of neuroscientists to the study of neurotrophins in depression as they are neuron survival factors of critical importance for the establishment and maintenance of neural circuits during development and in adult subjects [19–21]. 

Neurotrophnis constitute a family of 4 distinct secreted growth factors (nerve growth factor, NGF; brain-derived neurotrophic factor, BDNF; neurotrophin-3, NT-3; and neurotrophin-4, NT-4) which upon binding to membrane receptors in the target neurons activate an intracellular cascade which promotes survival and trophic effects. Each neurotrophin binds with high affinity to specific members of the tyrosine kinase receptor family (Trk receptors; NGF binds to TrkA, BDNF and NT-4 bind to TrkB and, NT-3 binds not only to TrkC but also to the other Trk receptors with low affinity). In addition, all the neurotrophins bind with low affinity to p75 (NTR), which not only is a very different receptor, responsible for storing and transporting neurotrophins, but also promotes neuronal cell death to sculpt neuronal circuits during development. 

Initial preclinical studies showing that expression of BDNF was downregulated in the dentate gyrus and hippocampus of rats subjected to chronic stress [22] have attracted interest of researchers on the potential involvement of BDNF in depression. Research accumulated in the last decade indicates that this neurotrophin is a central target in the pathogenesis of depression and suicidal behaviour [23, 24]. Expressions of BDNF, BDNF-regulated genes, and the receptor TrkB are decreased in postmortem brain samples from depressed humans [25] and in circulating lymphocytes of depressed patients during a drug-free period [26]. Consistent with these findings, serum levels of BDNF are also decreased in MDD patients [27, 28] and polymorphisms in the BDNF gene may be predictive of the chronicity of the disease [29]. Moreover, expression of BDNF is upregulated both in human and experimental animals by antidepressant treatments, including electroconvulsive therapy and repetitive transcranial magnetic stimulation [30–34]. In addition, BDNF (and also NT3) produced antidepressant effect on behavioral models of depression [35, 36] which are abolished in mice deficient in TrkB receptor [37]. Together these findings support a causal implication of BDNF in the genesis of MDD. Discrepancies present in the literature concerning the occasional absence of BDNF upregulation by different classes of antidepressants have been attributed to the route of administration, the doses of drugs employed, or, remarkably, a differential effect of the different antidepressants on the transcription on the four different exons present in the BDNF gene [23]. However, it must be mentioned that BDNF heterozygous knockout mice do not display anxious or depressive-like behaviors [38].

The expression of the BDNF receptor TrkB is also upregulated by chronic electroconvulsive seizure and antidepressant drug treatments [39]. Furthermore, increase of BDNF signaling by overexpression of the full length TrkB gene in mice results in an antidepressant-like behavioral response [40]. However, the implication of TrkB in depression pathophysiology bears more complexity because the TrkB gene in addition to the active full length isoform has a truncated isoform which modulates negatively BDNF signaling [23]. In fact, mutant mice with a forebrain directed deficiency in TrkB exhibit symptoms of attention-deficit disorder rather than depressive behaviour [41].

The formation of BDNF takes place by proteolytic cleavage of a larger precursor protein termed proBDNF. ProBDNF is able to bind the low-affinity receptor p75^NTR^ exerting an opposite effect to that of BDNF/TrkB signaling [42, 43]. Consistent with this finding, it has been found that the serum levels and the expression of both proBDNF and p75^NTR^ in circulating lymphocytes are up-regulated in MDD [44]. According to these facts it has been proposed that not only the expression of BDNF and TrkB but also the ratio between BDNF-TrkB and proBDNF-p75^NTR^ is dysregulated in MDD [44]. Evidence from a role of the p75^NTR^ receptor in depression is also supported by genetic evidence, because the missense Ser205Leu polymorphism of this gene appears to have a protective effect against the development of MDD in women [45]. 

The involvement of other neurotrophins and receptors in MDD has received less attention. Expressions of NT-3 and two members of the related family of glial cell line-derived neurotrophic factor, GDNF and ARTN, were found down-regulated in circulating blood cells of patients with MDD but not in bipolar disorder [46]. 

## 3. MicroRNAs and Depression

From the beginning of the present century, it was recognized that the genome, in addition to produced mRNA destined to form proteins which regulate cell function, generates also small units of noncoding RNA, termed microRNA (miRNA). miRNAs are regulatory molecules which control gene function by cleaving or repressing the translation of target mRNAs. miRNAs are very conserved among the different species and participate critically in most biological processes. Three aspects of miRNA is particularly relevant in medicine: (1) dysregulation of specific miRNAs are associated to many diseases; (2) levels of miRNAs can be identified and quantified by RT-PCR in the serum serving as biomarkers of different diseases; and (3) they can be silenced in vivo by administration of miRNAs inhibitors (antagomir) or employed as exogenous therapeutic agents to influence gene transcription and protein synthesis. 

miRNAs, as neurotrophnis, are involved in neuron survival, synaptogenesis, and neural plasticity, and their implication in psychiatric diseases is beginning to be explored (see [47]). Alterations of various miRNAs, including miR-30e, miR-182, and miR-132 have been implicated in MDD [47–50]. Remarkably, miR-132, and miR-182 regulate negatively the expression of BDNF and were found to show increased serum levels in MDD patients [50]. In preclinical studies, miR-212, which also regulates the expression of BDNF, was overexpressed in the dentate gyrus and serum after electroconvulsive stimulation [51]. Together these findings suggest that future functional studies of miRNA will provide significative advances in the understanding of psychiatric diseases including the design of novel treatments (see review [6]).

## 4. Stress Hormones and Depression

A large number of clinical and basic researches indicate that MDD is associated with a maladaptive response to stress, due to dysfunction of the hypothalamic-pituitary-adrenal axis (HPA axis) [52–54]. Abnormal hormone dynamics is a constant feature in mood disorders and can precede the onset of MDD [55] supporting the involvement of the HPA axis in this disease [56]. Stress hormonal alterations observed in MDD include impaired inhibition of cortisol release by dexamethasone, elevated cortisol values, increased excretion of cortisol and an overactive response to psychological stressors. Assays to evaluate HPA dysfunction, such as the dexamethasone suppression test or the dexamethasone/corticotropin releasing hormone test, have been useful to establish objective parameters in the diagnosis of endogenous mood disorders and to predict response to antidepressant treatment [57–59]. 

Regardless of whether these alterations are at the origin (i.e., Cushing disease) or are a consequence of MDD, it is important to remark that the elevated levels of stress and glucocorticoid hormones interfere with normal hippocampal neurogenesis [60] contributing to the development of the disease. Consistent with this interpretation, a glucocorticoid receptor target gene, the serum- and glucocorticoid-inducible kinase 1 (SGK1) which inhibits hippocampal neurogenesis, is upregulated in depressed patients and in animal models of depressive behavior [60]. In addition, there is evidence for a role of corticosteroids modifying the function of BDNF, suggesting a functional crosstalk between stress hormones and BDNF signaling of potential implication in the pathogenesis of MDD [61]. 

## 5. Inflammation and Depression

Evidence for immune system involvement in the pathophysiology of major depressive disorder is abundant and solid (see reviews [62–64]). As mentioned above, a characteristic feature observed in MDD patients is the elevation of glucocorticoids. However, in spite of the potent anti-inflammatory effect of glucocorticoids, MDD patients exhibit elevated levels of circulating proinflammatory cytokines, including interleukin-1, interleukin-6, tumor necrosis factor alpha, and some soluble interleukin receptors [28, 65–67]. The proinflammatory cytokines not only participate in the innate immune response and inflammation but also have important metabolic and endocrine effects including neurotransmitter metabolism, neuroendocrine function, and neural plasticity. Remarkably, administration of interleukin-6 induces depressive-like behaviors and neutralizes the antidepressant effect of fluoxetine in experimental animals [68]. In a similar fashion, people treated with inflammatory cytokines such as interferon alpha develop depression that is indistinguishable from depression in nonmedically ill populations (see [69]). Furthermore, expression of different cytokines and genes implicated in cell death is up-regulated in postmortem brain tissue of MDD patients suggesting local inflammatory, apoptotic, and oxidative stress in brain regions involved in reward-related behaviors [70]. The involvement of cytokines in behavior and in different functions of the nervous system is also sustained by the presence of specific receptors in hippocampus and hypothalamic nuclei [71]. Remarkably, proinflammatory cytokines stimulate the hypothalamic-pituitary-adrenal axis, activate the secretion of growth hormone, and inhibit thyroid-stimulating hormone secretion [72]. All these endocrine effects are associated with MDD.

A striking finding about the role of proinflammatory cytokines in MDD is that, in contrast with the elevated levels in the blood, the level of interleukin-6 in cerebrospinal fluid is reduced in MDD patients and the decreased level is predictive of future depression in old women [73]. 

## 6. Gut Microbiota and Depression

There is growing evidence for the occurrence of a functional interplay between gut microbiota and brain function (brain-gut axis). According to this view the microbiota can influence brain chemistry and consequently behavior. Consistent with this idea, it has been found that the composition of gut microbiota in animal models of depression and chronic stress shows differences with that of healthy animals [74]. Leptin [75], ghrelin [76], cholecystokinin [77], and other various factors are signaling peptides produced in the gastrointestinal system with a direct influence on the central nervous system, including modulation of neurogenesis, which might be implicated in MDD. However, at the present, we are still far from assigning a role for disbalances in the gut microbiota in the pathophysiology of depression and alterations in gut flora may be secondary to abnormal gastrointestinal dynamics in MDD patients. 

## 7. Concluding Remarks: Adult Neurogenesis and MDD

The evidence surveyed in this review supports a primary involvement of disturbed adult neurogenesis and altered synaptic connectivity in the origin of MDD (see reviews [11, 78]). In adult mammals, neurogenesis is sustained by two specialized niches of neural progenitors, the subventricular zone of the lateral ventricles and the subgranular zone of the hippocampal dentate gyrus [79]. Preclinical studies have shown that hippocampal neurogenesis is altered in chronic stress which is considered as an animal model of clinical depression [80]. In addition, most studies point to hippocampal neurogenesis as the target for antidepressant treatments [80–83]. As listed in this review, deficient neurogenesis may be caused by distinct primary alterations of a complex signaling network which includes, at least, the following players: dysfunction of the hypothalamic-pituitary-adrenal axis; deficient production of neurotrophins; abnormalities in the expression of MiRNAs; dysregulation of proinflammatory cytokines; and, even, the abnormal delivery of gastrointestinal signaling peptides. Any of these alterations appear to promote a similar phenotype characterized by major mood alteration. In addition, all of those factors are functionally interconnected in such a fashion that initial alteration in one of them results in abnormalities in the others. However, whether the reduced neurogenesis is the cause of MDD or whether neurogenesis is only necessary to ameliorate the disease needs to be clarified. Postmortem studies in humans have found no change in cell proliferation between major depression patients and control samples [84]. Furthermore, no depressive-like behavior was induced by experimental inhibition of cell proliferation in the hippocampus of animal models [85]. And, most striking, increased neurogenesis has been implicated in the induction of anxious behavior in mice questioning the simplistic view that more newborn neurons are always better for mental health [86]. A further explanation is that changes in adhesion molecules, like neural cell adhesion molecule (NCAN) associated with neurogenesis and also with synaptic plasticity, may play a central role in MOD [11].

Regardless of whether the role of hippocampal neurogenesis in MDD concerns the etiology or the antidepressant treatment, it is likely that future treatments of MDD will be designed to target neurogenesis and neural plasticity as a central factor in the pathogenesis of this disorder. Potential candidates for this purpose are different families of secreted factors with positive influence in neurogenesis. Remarkably, FGFs, which are a family of growth factors involved in the control of proliferation and neuroplasticity, have been recently implicated in the pathophysiology of MDD [87]. Several ligands of the FGF family, such as FGF-2, are expressed in the adult brain and become downregulated in individuals suffering from MDD [88]. In addition, exogenous FGF-2 has antidepressant effect on animal models of depressed behavior [89]. Vascular endothelial growth factor (VEGF) is an angiogenic growth factor which promotes hippocampal neurogenesis [90] implicated also in the pathophysiology of MDD [91]. SB100B is a protein associated with MDD which is expressed and secreted by glial cells and other nonneural cell lineages implicated in synaptogenesis and neuronal survival [92]. A number of recent studies have observed that SB100B is increased in the hippocampus, serum, and cerebrospinal fluid of MDD patients, most likely reflecting a response of glial cells to neural damage (see [93] and references therein) and also that the basal levels of serum may predict the outcome of the therapeutic response to antidepressants [94]. 
